# The Effects of a Novel *Astragalus*-Based Extract (Keyfobell Powder (KFB)) on Longitudinal Bone Growth via IGF-1 Upregulation: A Potential Growth Hormone Alternative

**DOI:** 10.3390/nu17030416

**Published:** 2025-01-23

**Authors:** Myong Jin Lee, Daesik Jeong, Ji Hwan Lee, Jaeha Kang, Jihye Choi, Jaeok Seo, Hong Il Kim, Jisoo Seo, Kiseong Ko, Dong Hyuk Nam, Hye Lim Lee, Ki Sung Kang

**Affiliations:** 1 Department of Preventive Medicine, College of Korean Medicine, Gachon University, Seongnam 13120, Republic of Korea; myongene@gachon.ac.kr (M.J.L.); kleert26@gmail.com (J.H.L.); 2 College of Convergence Engineering, Sangmyung University, Seoul 03016, Republic of Korea; jungsoft97@smu.ac.kr; 3 Department of Computer Science, Sangmyung University, Seoul 03016, Republic of Korea; ramg0710@5works.co.kr; 4 Chong Kun Dang (CKD) Pharm Research Institute, Yongin 16995, Republic of Korea; jihye.choi@ckdpharm.com (J.C.); jaeok@ckdpharm.com (J.S.); hongil@ckdpharm.com (H.I.K.); seojs@ckdpharm.com (J.S.); kiseong@ckdpharm.com (K.K.); namdh@ckdpharm.com (D.H.N.); 5 Department of Pediatrics, College of Korean Medicine, Daejeon University, Daejeon 34520, Republic of Korea

**Keywords:** *Astragalus* extract, growth hormone, ghrelin, insulin-like growth factor-1, insulin-like growth factor binding protein-3

## Abstract

Background/Objectives: This study evaluated the effects of a novel Astragalus extract (Keyfobell powder [KFB]) composed of *Astragalus membranaceus*, red ginseng (*Panax ginseng* C. A. Meyer), and *Cervi Parvum Cornu* as a potential growth hormone (GH) alternative. The primary focus was placed on its impact on longitudinal bone growth through the upregulation of circulatory insulin-like growth factor (IGF)-1. Methods: We performed in vitro and in vivo experiments using a hypothalamic cell line and Sprague–Dawley (SD) rats. Quantitative RT-PCR was performed to determine growth hormone-releasing hormone (GHRH) and ghrelin mRNA expressions in GT1-7 cells. The treatment groups were administered KFB at various dosages, and the positive controls received recombinant human GH. Body weight, bone length, and density were assessed, along with serum levels of insulin-like growth factor binding protein (IGFBP)-3 and IGF-1. Results: KFB and somatropin exhibited no cytotoxic effect in GT1-7 cells and increased GHRH and ghrelin mRNA levels in a dose-dependent manner. KFB administration resulted in a significant dose-dependent increase in body weight and bone growth (femur and tibia). Changes in IGF-1 and IGFBP-3 levels were comparable to those observed in the GH-treated group. Based on network pharmacological analysis, multiple compounds in KFB ((20S)-20-hydroxypregn-4-en-3-one, 2-isopropyl-3-methoxypyrazine, caproic acid, daidzein, furfuryl alcohol, lauric acid, octanal, and salicylic acid) may synergistically regulate the PI3K-Akt, Ras, and Rap1 signaling pathways linked to growth control and cartilage formation, leading to a possible increase in height. Conclusions: Our results suggest that KFB can function as a GH-mimetic agent that promotes bone growth through IGF-1 upregulation.

## 1. Introduction

Pediatric patients are often concerned when they experience short stature, which leads to evaluations by various healthcare professionals, including endocrinologists. When assessing children with significant height deficits, it is crucial to consider a range of underlying medical conditions, such as growth hormone deficiencies, genetic syndromes, and chronic diseases that can affect growth. Despite extensive scientific and clinical studies, children are often classified as having idiopathic short stature (ISS) during childhood. Children with growth hormone deficiency (GHD) exhibit shorter heights and slow growth rates as they progress through age and puberty [1].

The anterior pituitary gland in the brain produces the small growth hormone (GH) protein. The hypothalamus primarily controls the production and release of this hormone, which is influenced by the balance between somatostatin and growth hormone-releasing hormone (GHRH) [2]. Pulsatile GH secretion is affected by various factors, including age, sex, pubertal status, diet, physical activity, fasting period, sleep pattern, and body composition. Among these factors, ghrelin, the endogenous ligand for the GH secretagogue receptor [3], has been found in the hypothalamus in mRNA form despite being a peptide primarily produced in the stomach [4]. Studies have demonstrated that ghrelin potently stimulates GH secretion in humans [5]. GHD occurs when hypothalamic or pituitary disorders disrupt GH secretion [3]. A decline in GH secretion with age is associated with a decrease in growth hormone-releasing hormone (GHRH) secretion. Therefore, GH is widely used for the treatment of GHD or short stature; however, its therapeutic use can lead to adverse side effects, including cell proliferation, which may increase the risk of cancer [6]. In addition, GH therapy may induce other complications, such as diabetes, fluid retention, joint and muscle pain, and hypertension [6]. Owing to these effects, the application of GH remains limited, particularly in pediatric populations.

In light of these concerns, interest in alternative treatments derived from natural sources that present lower-risk profiles has increased. Certain Oriental medicinal herbs, such as *Phellodendron amurense*, have demonstrated potential as treatments for growth deficiencies [7]. In Korea, traditional herbal medicines are used to treat growth retardation in children [8]. In particular, *Astragalus* extract, composed of the stem of *Eleutherococcus senticosus* and the roots of *A. membranaceus* and *Phlomis umbrosa*, has been evaluated as a promising therapeutic candidate, as it may mimic the effects of GH without the associated risks using rats as well as humans [9,10,11]. The present study investigated the effects of a novel *Astragalus* extract (Keyfobell powder [KFB]), composed of roots of *A. membranaceus* and red ginseng (*P. ginseng* C. A. Meyer), and Cervi Parvum Cornu, a young horn of *Cervus elaphus* Linne and *Cervus nippon* Temminck on bone growth and development. These effects were compared with recombinant human growth hormone (somatropin) as a positive control, which is a synthetically produced form of human growth hormone that functions by replacing growth hormones in the body. It is used to promote body weight gain and growth in pediatric patients with specific conditions that inhibit normal growth and development.

Furthermore, this study aimed to bridge traditional knowledge and modern scientific approaches by verifying predicted pharmacological targets and pathways through a network pharmacological analysis. In addition, by exploring the growth-promoting potential of KFB, this study aimed to expand the scope of traditional herbal medicines in contemporary health applications, emphasizing a systematic and evidence-based approach to understanding and utilizing these ancient resources in modern health challenges.

## 2. Materials and Methods

### 2.1. Preparation and Analysis of Indicator Compounds in Astragalus Extract Mixture KFB

KFB extracts were prepared by Chong Kun Dang (Seoul, Republic of Korea) according to pharmaceutical standards. Briefly, *A. membranaceus* water extract, red ginseng (*P. ginseng* C. A. Meyer) 30% ethanolic extract, and *Cervi Parvum Cornu* 50% ethanolic extract were purchased from SUNGIL BIOEX Co., Ltd. (Hwasung, Republic of Korea). *A. membranaceus*, red ginseng extract, and Cervi Parvum Cornu extracts were mixed in a ratio of 1:1.1:7.4. After mixing 3 extracts, the concentrate was dried to prepare KFB, and HPLC analyses were conducted for the analysis of indicator substances.

**Figure 1:**
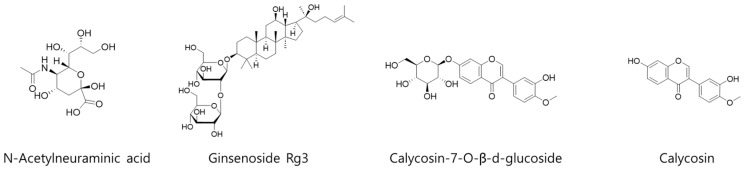
Chemical structures of 4 representative compounds (*N*-acetylneuraminic acid, ginsenoside Rg3, calycosin-7-O-β-d-glucoside, and calycosin) in KFB.

For quantitative analysis of 4 representative compounds (*N*-acetylneuraminic acid, ginsenoside Rg3, calycosin-7-O-β-d-glucoside, and calycosin) in KFB (Figure 1), 3 different analytical methods were applied. The details of each analysis method, equipment information, and column information are summarized in Table 1.

### 2.2. Cell Viability

Mouse hypothalamic GT1-7 cells were incubated in Dulbecco’s modified Eagle’s medium (DMEM) enriched with 10% fetal bovine serum (FBS), 100 U/mL streptomycin, and 100 U/mL penicillin. Culture conditions and cell viability were based on the method described by Lee et al. [12].

### 2.3. RNA Extraction and Real-Time Reverse Transcription-Polymerase Chain Reaction (RT-PCR)

Cells treated with KFB and somatropin were harvested after 24 h, and total RNA was extracted using a kit following the manufacturer’s protocol (Qiagen Inc., Valencia, CA, USA). The cDNA synthesis and PCR conditions were based on the method described by Lee et al. [13].

### 2.4. Animal Management and Administration

This study was approved by the Institutional Animal Care and Use Committee (IACUC) of the Advanced Medical Bio Research Institute and was conducted in accordance with relevant guidelines and regulations (Approval Number: 2023-1114-B, approved 11 November 2023), including criteria used for including and excluding animals. Thirty-five female Sprague–Dawley (SD) rats (3 weeks old) were obtained from Raon Bio (Yongin, Gyeonggi-do, Republic of Korea). The animal room breeding conditions were maintained at temperatures ranging 20–24 °C, humidity of 40–60%, negative pressure, and noise levels of <60 dB, with a 12 h light–dark cycle. A maximum of 4 rats were housed in a cage, and water and food were provided ad libitum.

Following a 1-week acclimatization period, the animals were randomly allocated as outlined in Table 2. To ensure unbiased allocation of animals into experimental groups, randomization was performed. Animals (35 SD rats) were assigned to the treatment and control groups using a random number tables. This process was performed prior to the start of the experiment by an independent individual not involved in the experimental procedures. The individuals administering the treatments were not involved in data collection or analysis. Similarly, the researchers responsible for measuring and recording outcomes were blinded to the group assignments of the animals. The G0, G1, G2, and G3 groups were administered orally daily at the doses outlined in Table 3 based on a dosage of 1 mL/100 g body weight, whereas the positive control group, P, received subcutaneous administration (S.C), with each group consisting of seven rats. The animal experimental schedule and sample doses used in this study were based on previous studies [10,11]. For daily S.C., the injection sites varied between the upper arm, thigh, and abdomen, with care taken to avoid repeated injections at the same site within a short period.

The body weights of the rats were measured and recorded at 0, 7, 14, 21, and 28 d after administration. In addition, the entire body lengths of the rats were photographed using a ruler at the same intervals to evaluate growth efficacy. At 28 d after administration, blood samples were collected under anesthesia with 3.0% isoflurane. Serum was separated and stored, and tissue autopsy was performed following euthanasia. The excised liver tissues were frozen and stored for genetic analysis. The excised bone tissue was preserved in 10% formalin solution, and tissue slides were prepared. The left leg was separated, and micro-computed tomography (CT) scans of the femur and tibia were conducted at the Seoul National University shared equipment facility.

### 2.5. Measurement of Femoral Tibial Length and Bone Analysis

The femur and tibia were examined using micro-CT before and 28 d after administration. Tibial microarchitecture, bone length, and mineral density were evaluated using μ-CT (SkyScan1272, Skyscan, Kontich, Belgium). The radiography source was configured at 60 kV, with an intensity of 166 μA and pixel size of 40 μm. To monitor changes in tibial length, the entire tibia was scanned in vivo under isoflurane anesthesia at the beginning of the study and on days 7 and 28. The volumetric bone mineral density (vBMD) and bone microarchitecture in the cancellous and cortical bones of the tibia were analyzed.

Ex vivo scanning was conducted using a 0.5 mm-thick aluminum filter. For the vBMD measurement, the scan was calibrated using phantoms with a vBMD of 0.25 and 0.75 g/cm^3^. Image analysis was performed by a medical professional (Oben Co., Ltd., Suwon, Gyeonggi-do, Republic of Korea).

### 2.6. Measurement of Bone Growth Plate Length

To assess the length of the bone growth plate, tetracycline hydrochloride (10 mg/kg) was administered intraperitoneally (I.P) 48 h before the completion of the experiment (28 d after the administration of the test substance). Upon completion of the experimental period, the mice were euthanized with CO_2_, and the proximal tibia was removed through laparotomy, fixed in 10% formalin solution, and subsequently decalcified. Frozen 40 μm-thick sections were prepared and examined using fluorescence microscopy.

### 2.7. Histological Evaluation of Bone Growth Factors

Histological evaluation was performed using fixed tissues, followed by decalcification, paraffin block production, and 3 μm-thick sectioning to generate tissue slides. Following hydration, the antigens were subjected to 10 mM sodium citrate buffer, and immunostaining was performed using antibodies (insulin-like growth factor [IGF]-1 and bone morphogenic protein-2 [BMP-2]). Subsequently, color development was performed using the Avidin/Biotin Complex (ABC) reagent and 3,3′-diaminobenzidine (DAB) solution, and counterstaining was performed using hematoxylin.

### 2.8. Analysis of Blood Growth Factors

Blood growth factor analysis in blood was conducted using serum IGF-1 and insulin-like growth factor binding protein (IGFBP)-3 enzyme-linked immunosorbent assay (ELISA) kits. The ELISA kits were used according to the manufacturer’s instructions. The concentration of each factor was calculated using a standard curve (R^2^ ≥ 0.95).

### 2.9. Network Pharmacological Analysis

Information on the chemical ingredients of red ginseng (*P. ginseng* C. A. Meyer, Ginseng Radix Rubra), *Astragali Radix*, and *Cervi Parvum Cornu* was manually collected from chemical compounds in the Northeast Asian traditional medicine (TM-MC 2.0) database [14]. Target identification was conducted for the selected compounds in KFB using curated data from TM-MC 2.0. From the 155 compounds analyzed, only those with blood–brain barrier permeability (BBB) marked as true, oral bioavailability (OB) marked as true, and a quantitative estimate of drug likeness (QED) of ≥0.35 were selected for target identification using prediction methods. The common targets were used to filter potential targets with an average of relevance score ≥ 1.65. These potential targets were then entered into the Search Tool for the Retrieval of Interacting Genes (STRING) database (https://string-db.org/) [15], accessed on 8 January 2021, to establish protein–protein interaction (PPI) relationships. The PPI network was constructed using the web version of Cytoscape (3.9.0, Seattle, WA, USA), and the topological features of the major targets were evaluated using three parameters: degree of ≥17, betweenness centrality of ≥0.01, and proximity centrality of ≥0.5. The Gene Ontology (GO) and Kyoto Encyclopedia of Genes and Genomes (KEGG) pathway enrichment analyses of the key targets were performed using Enrichr (https://maayanlab.cloud/Enrichr/), accessed on 13 September 2024 [16,17,18]. Pathways with *p* < 0.05 were considered significant, and the GO and KEGG pathway enrichment analysis results were illustrated through a web diagram. Finally, an integrated network of compounds, key targets, and pathways were analyzed and constructed using the web version of Cytoscape.

### 2.10. Statistical Analysis

All data were verified for statistical significance using SPSS software v.19.0 (IBM, New York, NY, USA). Normality of the data was assessed using the Shapiro–Wilk test, kurtosis, and skewness. The comparison of all evaluation results based on time points was performed using a paired t-test if normality was observed, and the Wilcoxon signed ranks test and post-hoc test (Bonferroni correction) were used if normality was not observed. Homogeneity was analyzed using the paired *t*-test (*p* > 0.05). Comparisons between groups was examined using repeated-measures (RM) analysis of variance (ANOVA) and analysis of covariance (ANCOVA) (*p* <0.05).

## 3. Results

### 3.1. Chromatography and Quantitation of the Four Compounds in KFB

**Figure 2:**
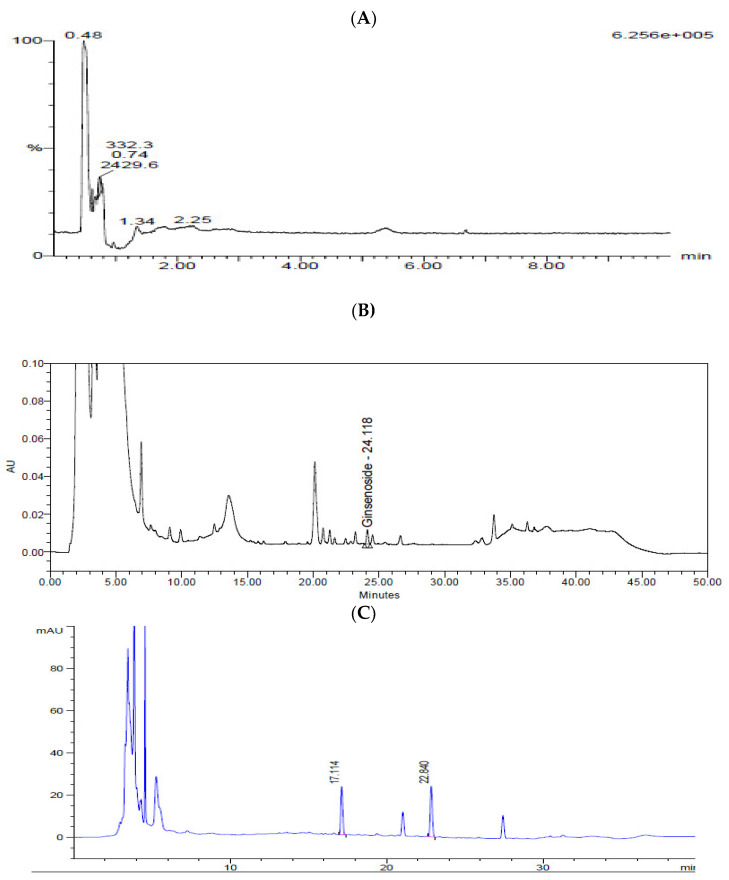
Chromatograms of KFB. (**A**) LC-MS chromatogram of *N*-acetylneuraminic acid. (**B**) HPLC chromatogram of ginsenoside Rg3. (**C**) HPLC chromatogram of calycosin-7-O-β-d-glucoside and calycosin.

*N*-Acetylneuraminic acid from Cervi Parvum Cornu, ginsenoside Rg3 from red ginseng, and calycosin-7-O-β-d-glucoside and calycosin from *A. membranaceus* were chosen as indicator substances of KFB (Figure 2). The contents of *N*-acetylneuraminic acid, ginsenoside Rg3, calycosin-7-O-β-d-glucoside, and calycosin were 7.0 ± 0.2, 64.3 ± 1.9, 6.8 ± 0.1, and 4.5 ± 0.1 μg/g dry extract, respectively.

### 3.2. Effect of KFB on Cytotoxicity in GT1-7 Cells

**Figure 3:**
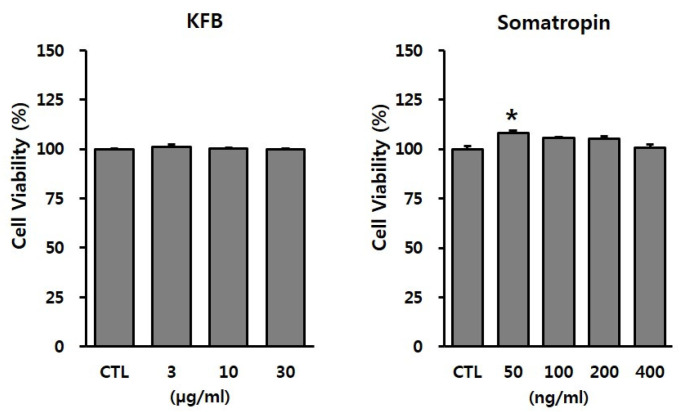
The effect of KFB on cell viability in GT1-7 cells. Cell viability was assessed using the EZ-Cytox assay, as discussed in the Materials and Methods section. All data were presented as percentages of the optical density of the control. Results are expressed as means ± SD (*n* = 3). Statistical significance was set at * *p* < 0.05 compared to control group.

To evaluate the potential toxicity of the test materials, GT1-7 cells were treated with the indicated concentrations of KFB (3, 10, or 30 µg/mL) and somatropin (50, 100, 200, or 400 ng/mL). As shown in Figure 3, KFB did not exhibit cytotoxicity up to 30 µg/mL, whereas somatropin, used as a positive control, presented no toxic effect within the range 50–400 ng/mL. These non-toxic doses were used in subsequent in vitro experiments.

### 3.3. Effect of KFB on mRNA Expression of GHRH and Ghrelin in GT1-7 Cells

**Figure 4:**
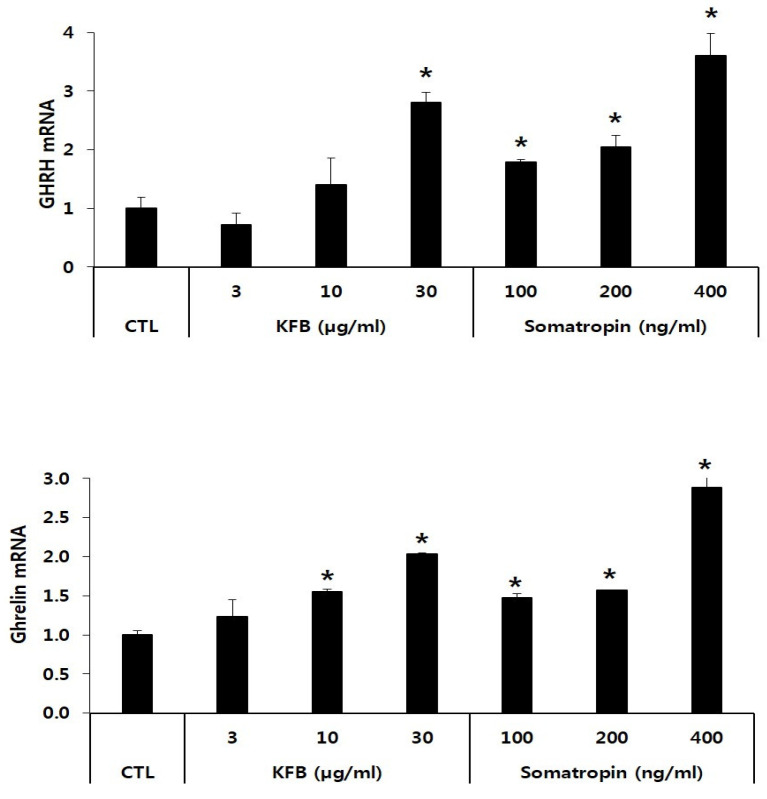
The effect of KFB on the gene expression of GHRH and ghrelin. The results are presented as means ± standard deviation (SD) (*n* = 3). Statistical significance was set at * *p* < 0.05, compared with the control group.

The expression of GHRH and ghrelin mRNA was significantly increased in KFB- and somatropin-treated GT1-7 cells in a concentration-dependent manner relative to that in the control. The GHRH mRNA level showed a 2.8-fold increase at 30 µg/mL of KFB compared to that of the control, whereas the expression of somatropin mRNA exhibited the highest value (3.6-fold that of the control) at 400 ng/mL somatropin among the three groups (control, KFB, and somatropin). Similarly, in comparison to the control, ghrelin mRNA expression was upregulated in the two groups, exhibiting an increase that correlated with the treatment concentration (Figure 4).

### 3.4. Effect of KFB on Changes in Body Weight and Longitudinal Bone Growth

**Figure 5:**
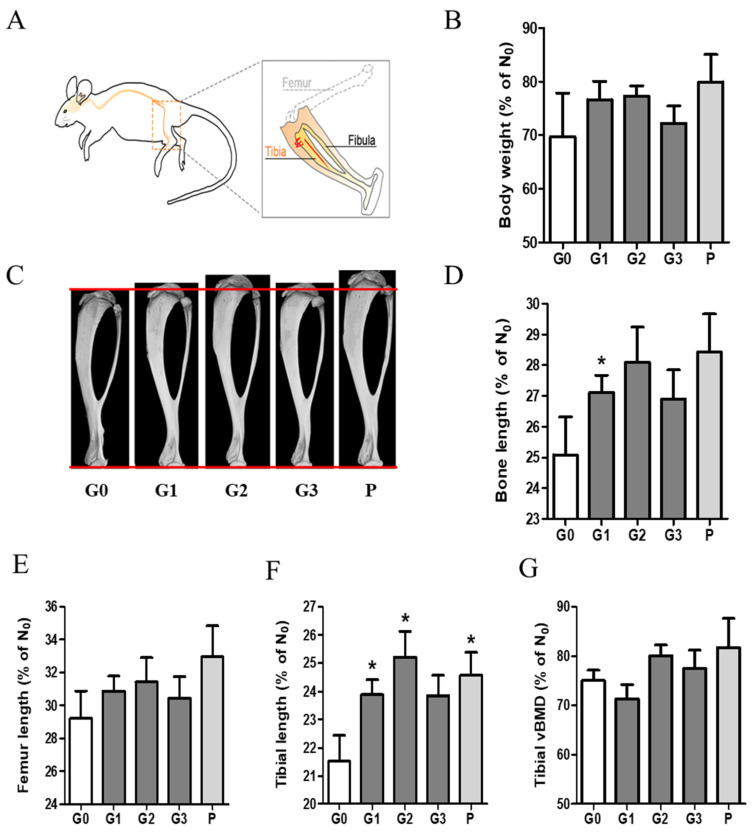
The effect of KFB on body weight and bone growth. (**A**) The bone schematic diagram, (**B**) the body weight ratio, (**C**) a 3D image showing tibial length, (**D**) the bone length ratio, (**E**) the femur length ratio, (**F**) the tibial length ratio, and (**G**) the tibial vBMD. G0, non-treated group; G1, 304 mg/kg KFB in milk; G2, 2940 mg/kg KFB in milk; G3, 304 mg/kg KFB in saline; and P, 0.2 mg/kg rhGH. * *p* < 0.05 vs. G0 using RM-ANOVA and ANCONA (*n* = 7). D_0_ is a time point before sample administration. vBMD, volumetric bone mineral density.

To investigate the effect of KFB on body weight and bone growth, we measured and analyzed the body weight and bone ratios in group 6, as shown in Figure 5. The ratio (%) shown in the graph was calculated based on a comparison with the time point (D_0_) before the administration of each sample. Body weight increased in all the groups. Notably, the G1 (304 mg/kg KFB in calcium milk), G2 (2940 mg/kg KFB in calcium milk), and P (0.2 mg/kg rhGH) groups showed a higher tendency than the control group. We verified the efficacy of KFB by measuring the bone, femur, and tibial lengths. Furthermore, the tibial length represented three dimensions (Figure 5C), similar to body weight and length. Body weight was consistent with bone, femur, and tibial lengths. Bone growth, including bone, femur, and tibial lengths, positively correlated with body weight (Figure 5B–F). The addition of calcium milk did not show a significant difference in bone length, but a slight increase in BMD was observed (Figure 5G).

### 3.5. Effects of KFB on Growth Plate and Microstructure of Cortical and Trabecular Bones

**Figure 6:**
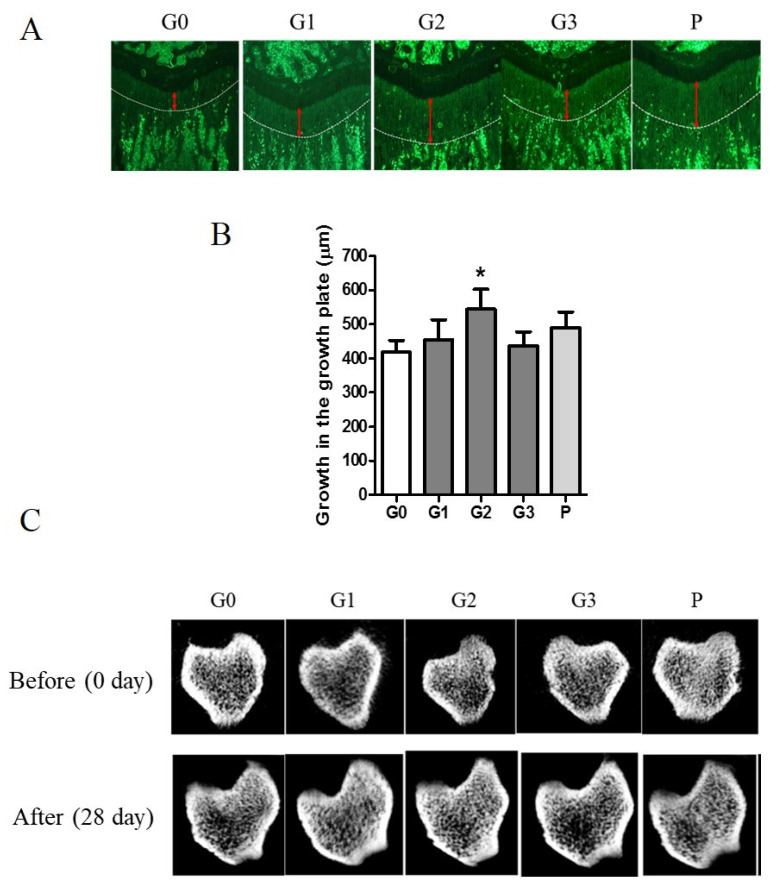
The effect of KFB on the growth plate and microstructure of the cortical and trabecular bones. (**A**) The fluorescent image of the growth plate. Red arrows indicate the length of the growth plate. (**B**) The numerical values of the growth plate. (**C**) The microstructure of the cortical and trabecular bones. G0, non-treated group; G1, 304 mg/kg KFB in milk; G2, 2940 mg/kg KFB in milk; G3, 304 mg/kg KFB in saline; and P, 0.2 mg/kg rhGH. * *p* < 0.05 vs. G0 using RM-ANOVA and ANCONA (*n* = 7). The length of bone growth is denoted by a double-headed red arrow.

Tetracycline, a fluorescent marker, was used to measure growth plate length by labeling the bone beneath the growth plate of the proximal tibia. The length of bone growth is denoted by a double-headed red arrow and represents numerical data (Figure 6A). Compared with the control group, the G2 and P groups exhibited a >30% expansion of the growth plate (Figure 6B). The control group demonstrated a 74.98% change in vBMD, whereas the G2 group exhibited a significant increase of 79.94% after 28 d. The G2 group exhibited an enhanced microstructure of the cortical and trabecular bones (Figure 6C).

### 3.6. Effects of KFB on Growth Factor Expression in Blood and Bone Tissues

**Figure 7:**
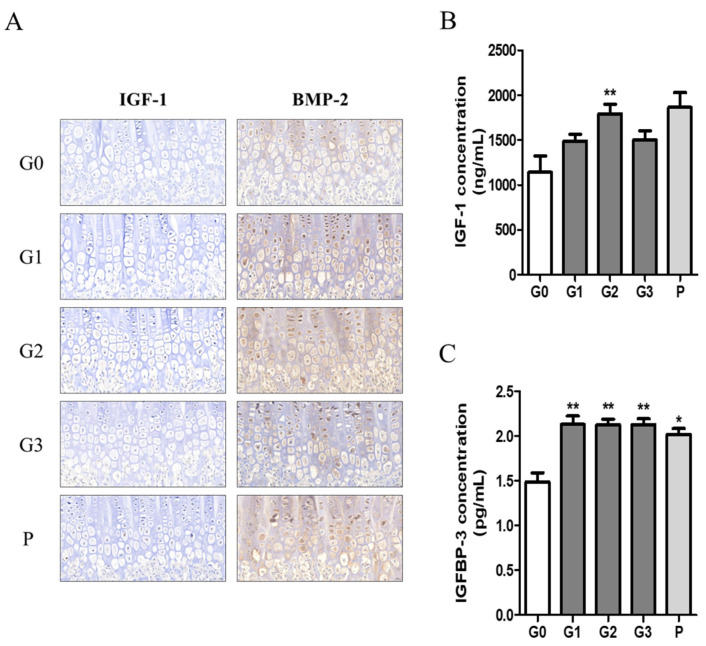
The effect of KFB on growth factors. (**A**) Staining of growth factors IGF-1 and BMP-2 in bone tissues, (**B**) growth factor IGF-1 concentration in blood, and (**C**) growth factor IGFBP-3 concentration in blood. G0, non-treated group; G1, 304 mg/kg KFB in milk; G2, 2940 mg/kg KFB in milk; G3, 304 mg/kg KFB in saline; and P, 0.2 mg/kg rhGH. ** *p* < 0.05 vs. G0 and * *p* < 0.05 vs. G0 using RM-ANOVA and ANCONA (*n* = 7).

We investigated growth factors such as IGF-1 and BMP2 in bone tissue using immunohistochemistry and quantified the expression levels of IGF-1 and IGFBP-1 in serum using ELISA. The expression levels of IGF-1 and BMP-2 tended to be higher in the hypertrophic zones of the G1, G2, and P groups (Figure 7A). Moreover, serum IGF-1 concentration was significantly higher in the G2 group, with levels comparable to those observed in the P group (Figure 7B). In addition, serum IGFBP-3 levels were significantly elevated in all experimental groups compared with those in the control group (Figure 7C).

### 3.7. Prediction of Molecular Mechanisms of KFB Using Network Pharmacology Analysis

**Figure 8:**
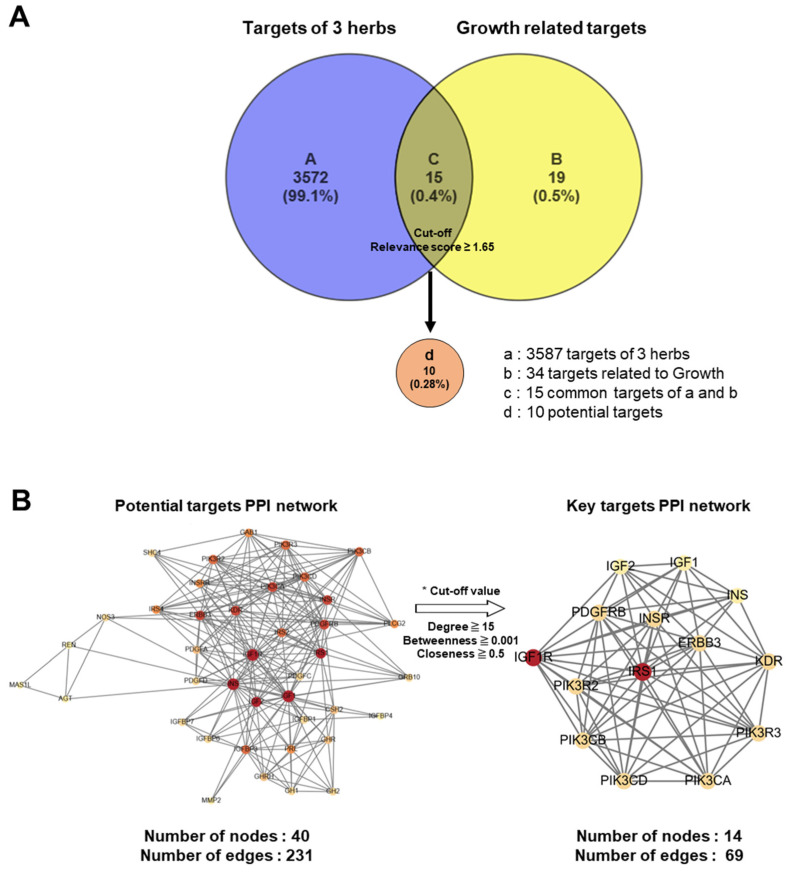
Network pharmacological analysis of KFB. (**A**) Venn diagram for target genes of 3 herbs (KFB) and growth-related target genes. (**B**) PPI networks of 40 potential targets (left) and 14 key targets.

Advances in network pharmacological analysis have provided opportunities to predict the unidentified molecular mechanisms of traditional herbal medicines, thereby highlighting the integrated influence of multiple active ingredients across various biological pathways [19,20]. In the present study, we conducted a network pharmacological analysis of KFB to investigate its components related to height growth. A curated resource was designed in the TM-MC 2.0 database for such purposes, comprising 155 chemical compounds of the four herbs and their 3587 associated target genes. In this analysis, 34 genes associated with height growth were extracted from the CTDbase database [21], and 15 genes were observed to be common targets between the four herbs and growth. Among these 15 common target genes, the 10 that satisfied the cut-off values (relevance score ≥ 1.65) were selected as potent targets (Figure 8A). A PPI network was analyzed and constructed to identify key targets among potential candidates (Figure 8B).

**Figure 9:**
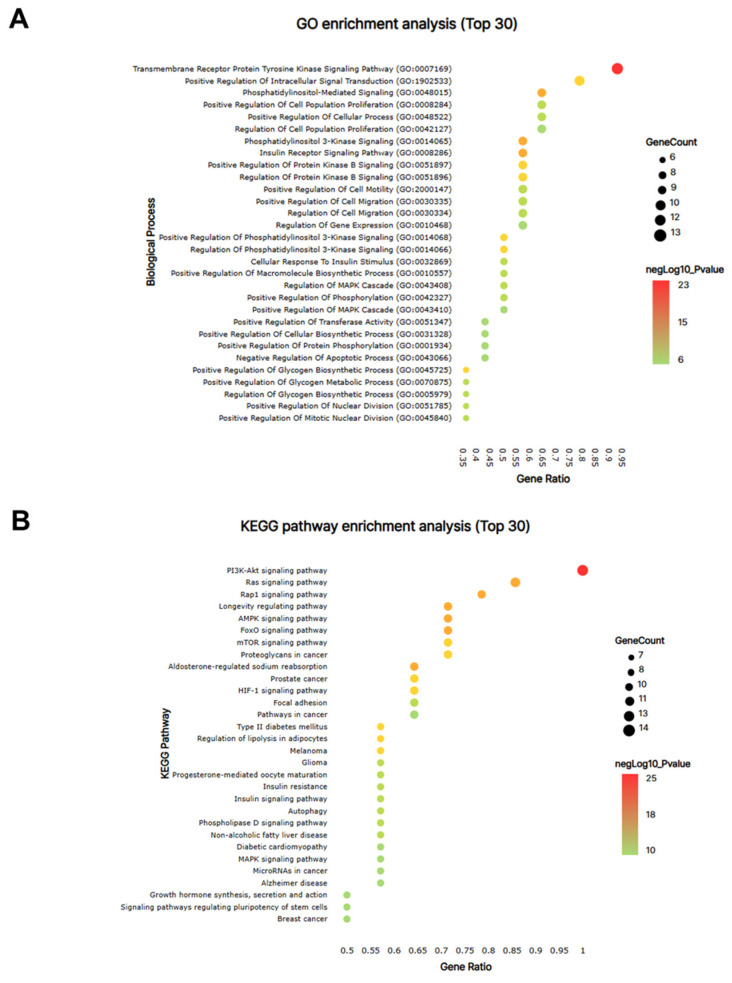
The network pharmacological analysis of KFB. The bubble map of GO (**A**) and KEGG pathway (**B**) enrichment analyses for the 14 key targets. Bubble size indicates the number of enriched genes, and bubble color difference shows the importance of target gene enrichment.

From the PPI network of 10 potential targets, 14 key targets were identified among 40 nodes that satisfied the degree, betweenness, and closeness criteria (Table 4). In addition, pathway enrichment analyses based on KEGG and GO for the 14 key target genes were performed (Figure 9A,B), revealing that the PI3K-Akt, Ras and Rap1 signaling pathways were among the top three. This indicates a strong link between growth control and cartilage formation, suggesting a possible increase in height.

IGF1, insulin-like growth factor I; IGF2, insulin-like growth factor II; ERBB3; receptor tyrosine-protein kinase erbB-3; INSR, insulin receptor; INS, insulin; IRS1, insulin receptor substrate 1; IGF1R, insulin-like growth factor 1 receptor; PIK3CA, phosphatidylinositol 4,5-bisphosphate 3-kinase catalytic subunit alpha isoform; VEGFR2, vascular endothelial growth factor receptor 2; PDGFRB, platelet-derived growth factor receptor beta; PIK3CB, phosphatidylinositol 4,5-bisphosphate 3-kinase catalytic subunit beta isoform; PIK3CD, phosphatidylinositol-4,5-bisphosphate 3-kinase catalytic subunit delta; PIK3R2, phosphatidylinositol 3-kinase regulatory subunit beta; PIK3R3, phosphatidylinositol 3-kinase regulatory subunit gamma.

**Figure 10:**
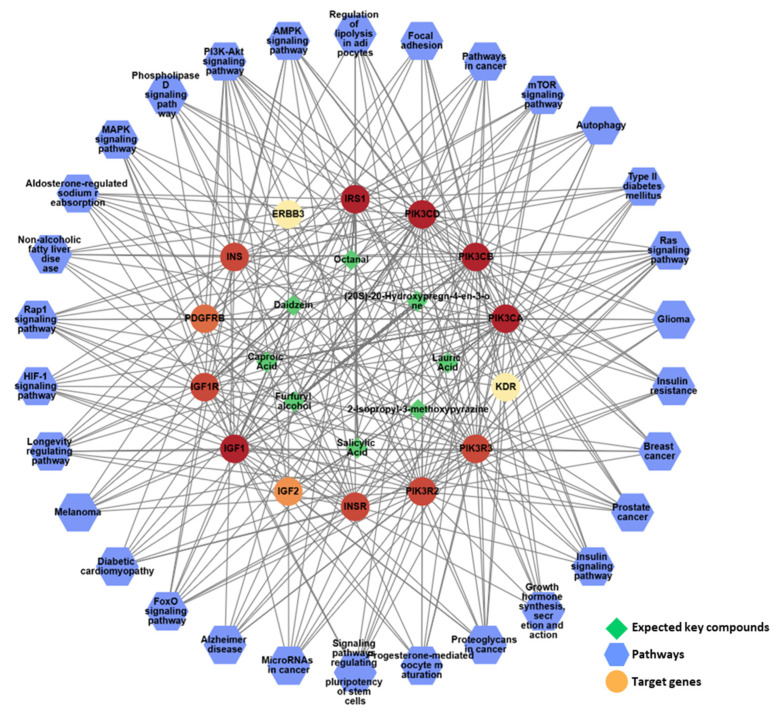
The network pharmacological analysis of KFB. The active C-T-P network. Green edges represent the correlation with the highest degree of the pathway and its targets.

To gain a comprehensive understanding of network pharmacology, a compound–target–pathway (C-T-P) network was constructed to analyze the intricate relationships between the different categories (Figure 10). The analysis of this C-T-P network revealed 52 nodes, consisting of eight key compounds, 14 key targets, and 30 pathways, interconnected by 477 edges that represent their interactions, further elucidating the underlying pharmacological mechanisms. Eight compounds in KFB ((20S)-20-hydroxypregn-4-en-3-one, 2-isopropyl-3-methoxypyrazine, caproic acid, daidzein, furfuryl alcohol, lauric acid, octanal, and salicylic acid) synergistically regulated the PI3K-Akt, Ras, and Rap1 signaling pathways, which are linked to growth control and cartilage formation (Figure 10, Table 5).

## 4. Discussion

Global research attention is increasingly being focused on growth retardation, as it is recognized as an important public health issue [1]. Children with short stature are often treated with growth hormone therapy, which is considered relatively safe and moderately effective for increasing height [22]. However, numerous risks are associated with GH treatment, including idiopathic diseases, hypertension, diabetes mellitus, cancer, and cardiovascular and joint issues. These health risks necessitate a critical re-evaluation of conventional GH treatments to discover potentially safer alternatives, particularly in pediatric cases where long-term effects remain unclear.

White and red ginseng is celebrated for its ability to increase energy levels and improve systemic health, which are critical factors for promoting growth. *Astragali Radix*, known for its immune-boosting and anti-aging effects, supports the metabolic processes essential for growth. *Cervi Parvum Cornu*, a valuable source of minerals and peptides, is traditionally used to enhance bone strength and development. The synergistic effects of these herbs on growth processes are hypothesized to involve the modulation of hormonal pathways, enhancement of nutritional absorption, and improvement of systemic metabolic efficiency.

In the present study, KFB significantly increased GHRH mRNA expression in KFB-treated hypothalamic neuronal cells. Similar to GHRH, ghrelin mRNA levels increased in GT1-7 cells following KFB treatment in a concentration-dependent manner. Both KFB and somatotropin significantly upregulate growth-associated gene expression. Furthermore, KFB treatment inhibited GnRH and netrin-1 mRNA expression in GT1-7cells, whereas somatotropin treatment increased their expression (Supplementary Figure S1). The contrasting effects of KFB and somatotropin on GnRH and its expression in GT1-7 cells highlight the intricate regulation of neuroendocrine signaling. The inhibitory role of KFB may indicates its potential as a suppressive agent against precocious puberty. These findings indicate that KFB can directly influence GnRH transcriptional activity, potentially enhancing the regulatory pathways of GHRH and ghrelin in the thalamus with potentially fewer side effects.

Based on the in vitro results, a 4-week KFB administration in vivo led to notable increases in body weight, femoral and tibial lengths, and vBMD, reflecting growth rates similar to those of the rhGH-positive control. In addition, KFB administration enhanced the growth plate length and density in the proximal tibial metaphysis. These outcomes align with the observed increase in serum IGF-1 and IGFBP-1 levels, both of which play vital roles in the promotion of skeletal development. IGFs and IGFBP-1 function as critical growth-promoting peptides [23]. Although the accurate measurement of serum GH is challenging owing to its pulsatile release and rapid clearance, serum IGF-1 concentrations are considered reliable indicators of GH activity. In our study, KFB administration resulted in elevated IGF-1 levels comparable to those achieved by rhGH, with even higher levels of IGFBP-3 in the KFB groups than in the control group. The increased IGF-1 and IGFBP-3 levels suggest the potential of KFB to induce a GH-like effect without the adverse outcomes associated with GH therapy. In addition, KFB enhanced the expression of BMP-2 in bone tissues. BMPs are members of the transforming growth factor-β superfamily that are essential for both embryonic development and adult bone maintenance. These proteins play crucial roles in bone remodeling, a continuous and essential process within the mature skeletal system [24,25]. The observed increases in IGF-1, IGFBP-3, and BMP-2 levels after KFB administration demonstrated its multifaceted effect on bone growth and suggested it as a promising natural alternative to conventional GH therapy.

Considering the challenges of elucidating the complex interactions within herbal medicines, a network pharmacological approach was used to investigate the underlying molecular mechanisms through which these herbs may contribute to growth. In the study of natural products, there are cases where multiple components exert effects on multiple targets rather than individual components, making it difficult to predict their mechanism of action. This method allows for a comprehensive analysis of the multi-target, multi-pathway effects of herbal constituents, offering insights into their potential synergy. By integrating bioinformatics, systems biology, and pharmacology, network pharmacology provides a robust framework for identifying key bioactive compounds and their target proteins as well as the signaling pathways they influence. In the present study, we tried to predict the synergistic mechanism of multiple compounds in KFB by using network pharmacology. As a result, eight compounds in KFB ((20S)-20-hydroxypregn-4-en-3-one, 2-isopropyl-3-methoxypyrazine, caproic acid, daidzein, furfuryl alcohol, lauric acid, octanal, and salicylic acid) may synergistically regulate the PI3K-Akt, Ras, and Rap1 signaling pathways linked to growth control and cartilage formation, thereby showing a possible increase in height.

Several studies have demonstrated that natural substances can enhance bone elongation. Recent studies on natural products as alternatives to growth hormone therapy have shown their promise, particularly for stimulating growth factors with fewer adverse effects. Therefore, we selected the *Astragalus* mixture KFB, which includes red ginseng and *Cervi Parvum Cornu*, for its potential to promote bone development and support growth. In adolescent rats, velvet antlers stimulate longitudinal bone growth and increase the growth plate by enhancing BMP-2, osteogenic activities, and the expression of bone matrix genes [26]. Red ginseng, a widely recognized herbal remedy in Asia, contains various biologically active substances such as glycans, peptides, and ginsenosides [27]. Ginsenosides, the active compounds in ginseng, have been shown to enhance growth rates in animal models by stimulating IGF-1 levels without having significant adverse effects [28,29]. These compounds are analogous to KFB and activate pathways associated with growth hormone secretion [30]. Previous studies have indicated that an *Astragalus* mixture increased IGF-1, IGFBP-3, and BMP-2 levels, which is consistent with our findings regarding KFB [10,31,32]. Similarly, the osteogenic properties of Rhizoma Drynariae extract have been investigated and showed a significant increase in bone mineral density in animal models [33]. This extract seemingly modulated the BMP pathway and enhanced IGF-1 expression [34], which are comparable to the effects observed when using KFB. Cumulative evidence from these studies highlights the potential of natural products to stimulate bone growth and promote longitudinal development, thereby supporting the hypothesis of viable herbal therapies.

Despite the promising findings of this study, this study has several limitations. First, the mechanism of the active component, which is critical for elucidating its influence on growth, has not been thoroughly investigated. Second, although this study focused on height growth in children, all experiments were performed using a mouse model, which may limit the direct application of the results to the human population. Although animal models provide valuable insights, the physiological differences between mice and humans should be considered when applying these findings to clinical settings. Third, while a network pharmacological analysis can provide valuable information [35], many issues need to be resolved for its validation, such as considering the actual content of each ingredient, various mixing ratios, and implementation in animal testing [36]. Further studies are required.

## 5. Conclusions

KFB increases the transcription of GHRH and ghrelin in hypothalamic cells. KFB administration resulted in a notable dose-dependent increase in body weight and bone growth (femur and tibia) in vivo. The observed changes in IGF-1 and IGFBP-3 levels were comparable to those observed in the GH-treated group. These findings indicate that KFB may function as a GH-mimetic substance that stimulates bone development by increasing IGF-1 production. Consequently, KFB has the potential to support children with idiopathic short stature or GH deficiency.

## Figures and Tables

**Table 1:** Quantitative analysis methods for *N*-acetylneuraminic acid, ginsenoside Rg3, calycosin-7-O-β-d-glucoside, and calycosin in KFB.

	*N*-Acetylneuraminic Acid	Ginsenoside Rg3	Calycosin-7-O-β-d-glucoside and Calycosin
LC System	Waters Acquity UPLC I-class (Waters Corporation, Milford, MA, USA)	Waters e2695 Separation Module (Waters Corporation, Milford, MA, USA)	Agilent 1260 Infinity (Agilent Technologies, Inc., Hachioji City, Tokyo, Japan)
Detector	Waters TQ-S micro	Waters 2489 UV/Vis Detector	Agilent 1260 DAD
Column	Waters Acquity UPLC CSH C18 130 Å column (1.7 μm, 2.1 × 50 mm)	GL Sciences Inertsil ODS-4 C18 100 Å column (5 μm, 4.6 × 250 mm)	YMC-Pack ODS-AM C18 120 Å column (5 μm, 4.6 × 250 mm)
Column Temp.	20 °C	25 °C	35 °C
Sample Temp.	15 °C	25 °C	15 °C
Detection	ESI+ SIR Mode	203 nm	254 nm
Flow rate	0.2 mL/min	1.0 mL/min	0.8 mL/min
Injection	10 μL	20 μL	10 μL
Mobile Phase	A: Methanol B: Water (0.1% Formic acid)	A: Acetonitrile B: Water	A: Acetonitrile B: Water
Gradient condition	Time A(%) B(%)	Time A(%) B(%)	Time A(%) B(%)
	0 20 80	0 30 70	0 10 90
	10 20 80	5 30 70	5 10 90
		20 50 50	25 50 50
		30 50 50	26 75 25
		32 70 30	30 75 25
		40 70 30	31 10 90
		42 30 70	40 10 90
		50 30 70	

**Table 2:** List of real-time RT-PCR primers used in this study.

Gene	Primer (5′-3′)
GHRH Ghrelin	F: CTCTGGGTGCTCTTTGTGA
	R: GAGTTTCCTGTAGTTGGTGGT
	F: GCTGTCTTCAGGCACCATCT R: GTGGCTTCTTGGATTCCTTTC
β-actin	F: CACCCGCGAGTACAACCTCC
	R: CCCATACCCACCATCACACC

**Table 3:** Administration information.

Name	Group	Dose	Usage
G0	Vehicle	Saline (*n* = 7)	QD ^1^/P.O ^2^
G1	Test	304 mg/kg KFB (solvent: calcium milk, *n* = 7)	QD/P.O
G2		2940 mg/kg KFB (solvent: calcium milk, *n* = 7)	QD/P.O
G3		304 mg/kg KFB (solvent: saline, *n* = 7)	QD/P.O
P	Positive control	0.2 mg/kg rhGH ^4^ (solvent: saline, *n* = 7)	QD/S.C ^3^

^1^ QD, once daily; ^2^ P.O, per os (oral administration); ^3^ S.C, subcutaneous injection; and ^4^ rhGH, recombinant human growth hormone.

**Table 4:** Key targets of KFB based on PPI network topological analysis.

No.	Uniprot ID	Gene	Relevance Score	Degree
1	P05019	IGF1	26.27	27
2	P01308	INS	20.33	26
3	P08069	IGF1R	11.06	26
4	P42336	PIK3CA	8.87	17
5	P35568	IRS1	7.31	20
6	P35968	KDR	7.07	17
7	P09619	PDGFRB	6.83	17
8	P01344	IGF2	5.72	18
9	P21860	ERBB3	3.01	16
10	P06213	INSR	1.80	16
11	O00329	PIK3CD	0.79	15
12	P42338	PIK3CB	0.37	15
13	O00459	PIK3R2	0.26	15
14	Q92569	PIK3R3	0.24	15

**Table 5:** Chemical information and C-T-P analysis of authentic key compounds in KFB.

Peak No.	Compound Name	Molecular Formula	Degree in Network	Correlating Targets	Origin
1	(20S)-20-Hydroxypregn-4-en-3-one	C_21_H_32_O_2_	1	*IGF1*	Ginseng Radix
2	2-Isopropyl-3-methoxypyrazine	C_8_H_12_N_2_O	1	*IGF2*	Ginseng Radix
3	Caproic acid	C_6_H_12_O_2_	1	*PIK3CA*	Ginseng Radix, Ginseng Radix Rubra
4	Daidzein	C_15_H_10_O_4_	2	*IGF1* *IGF1R*	Astragali Radix
5	Furfuryl alcohol	C_5_H_6_O_2_	2	*PIK3CB* *PIK3CD*	Ginseng Radix, Ginseng Radix Rubra
6	Lauric acid	C_12_H_24_O_2_	1	*PIK3CA*	Ginseng Radix
7	Octanal	C_8_H_16_O	1	*IRS1*	Ginseng Radix, Ginseng Radix Rubra
8	Salicylic acid	C_7_H_6_O_3_	1	*IRS1*	Ginseng Radix

## Data Availability

No data were used for the research described in the article.

## Supplementary Materials

The following supporting information can be downloaded at https://www.mdpi.com/article/10.3390/nu17030416/s1, Supplementary Figure S1: Effect of KFB on GnRH and Netrin-1 gene expression.
