# Supplementary material for: The Effects of a Novel Astragalus-Based Extract (Keyfobell Powder (KFB)) on Longitudinal Bone Growth via IGF-1 Upregulation: A Potential Growth Hormone Alternative
Source: Nutrients. 2025 Jan 23;17(3):416. doi: 10.3390/nu17030416 (PMC11820268; doi:10.3390/nu17030416)

Figure S1: The effect of KFB on gene expression of GnRH and Netrin-1

The production and secretion of gonadotrophin-releasing hormone (GnRH) are regulated by gonadal steroids via a negative feedback system. The mRNA expression of GnRH and Netrin-1 was evaluated to determine whether KFB regulates these factors in Hypothalamic GT1-7 cells that secrete GnRH. KFB inhibited the releases of GnRH and Netrin-1 compared with the control.

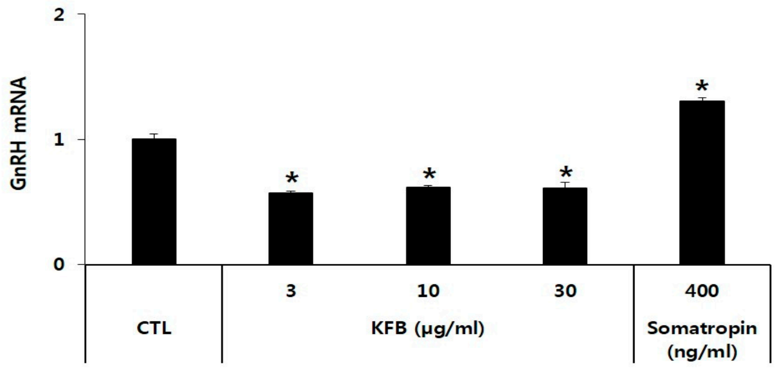

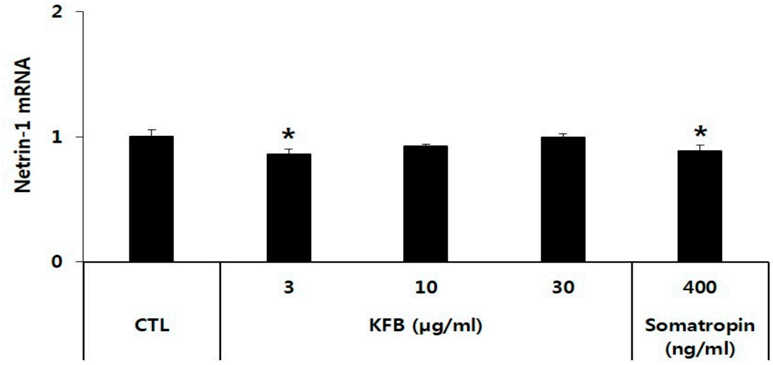

Supplement: Supplementary file 1 [file nutrients-17-00416-s001.zip › nutrients-3375863-Supplementary material.pdf]
